# Supplementary material for: From sequence to enzyme mechanism using multi-label machine learning
Source: BMC Bioinformatics. 2014 May 19;15:150. doi: 10.1186/1471-2105-15-150 (PMC4229970; doi:10.1186/1471-2105-15-150)
Supplement: Additional file 2 — Java code of ml2db. Additional file ml2db_code.tar.gz contains the Java source code to run the multi-label machine learning experiments and save the results to database. The code’s Javadoc is included. [file 1471-2105-15-150-S2.zip › additional file 2/ml2db/utils/doc/uk/ac/standrews/utils/main/maputils/OneToManyMap.html]

OneToManyMap


JavaScript is disabled on your browser.


- Overview
- Package
- Class
- Use
- Tree
- Deprecated
- Index
- Help

- Prev Class
- Next Class

- Frames
- No Frames

- All Classes

- Summary:
- Nested |
- Field |
- Constr |
- Method

- Detail:
- Field |
- Constr |
- Method


uk.ac.standrews.utils.main.maputils

## Class OneToManyMap<T,U>

- java.lang.Object
- - java.util.AbstractMap<K,V>
  - - java.util.TreeMap<T,U>
    - - uk.ac.standrews.utils.main.maputils.OneToMany<T,java.util.Set<U>>
      - - uk.ac.standrews.utils.main.maputils.OneToManyMap<T,U>

- All Implemented Interfaces:
  :   java.io.Serializable, java.lang.Cloneable, java.util.Map<T,java.util.Set<U>>, java.util.NavigableMap<T,java.util.Set<U>>, java.util.SortedMap<T,java.util.Set<U>>

  ---

    

  ```
  public class OneToManyMap<T,U>
  extends OneToMany<T,java.util.Set<U>>
  ```

  A map containing for each key a set of values. No key can be null. At most
  one key can be an empty-string. No value can be null (the Java TreeSet class
  used uses "null" as a return message when there is \*no\* mapping for a given
  key!). A value can be an empty string.

  Version:
  :   3 Jun 2008

  Author:
  :   Luna De Ferrari luna.deferrari-at-ed.ac.uk

  See Also:
  :   Serialized Form

- - ### Nested Class Summary

    - ### Nested classes/interfaces inherited from class java.util.AbstractMap

      `java.util.AbstractMap.SimpleEntry<K,V>, java.util.AbstractMap.SimpleImmutableEntry<K,V>`
  - ### Constructor Summary

    Constructors

    Constructor and Description

    `OneToManyMap()`

    Initialise an empty map

    `OneToManyMap(java.util.Vector<T> keys,
    java.util.Vector<U> values)`

    Initialise with a list of key-value pairs
  - ### Method Summary

    Methods

    | Modifier and Type | Method and Description |
    | --- | --- |
    | `void` | `addAll(java.util.Map<T,U> map)` |
    | `void` | `addAll(OneToManyMap<T,U> mapToAdd)` Add all elements of a one2many map to another map |
    | `boolean` | `addKeyAndValue(T key, U value)` Add a key-value pair to the map |
    | `void` | `clear()` |
    | `boolean` | `containsKey(java.lang.Object key)` Checks if the map contains a key |
    | `boolean` | `containsKeyValue(T key, U value)` Checks if the map contains a specific key-value pair |
    | `boolean` | `containsValue(java.lang.Object value)` Checks if the map contains a value |
    | `boolean` | `deleteKeyValue(T key, U value)` |
    | `java.util.Set<U>` | `generateAKeyValues(T key)` |
    | `java.util.Set<U>` | `getAKeyValues(T key)` Get the set of unique values for this key. |
    | `java.util.Vector<java.util.Vector>` | `getKeysVectorValuesVector()` Get 1. |
    | `java.util.TreeSet<T>` | `getKeysWithValue(U value)` Returns all the keys having a certain value |
    | `int` | `getKeyValuesPairsSize()` Get the number of unique key-value pairs in the map. |
    | `java.util.Set<T>` | `getNonEmptyKeys()` Get the set of unique, non-empty-string keys for this map. |
    | `int` | `getNonEmptyKeyValuesPairsSize()` Get the number of unique, non-empty key-value pairs in the map. |
    | `java.util.Set<U>` | `getNonEmptyValues()` Get the set of unique, non-empty-string values for this map. |
    | `U` | `getRandomValue(T key)` Get randomly one of the values of this key |
    | `java.util.Set<U>` | `getValues()` Get the set of unique values for this map. |
    | `static OneToManyMap<java.lang.String,java.lang.String>` | `invertKeysAndValues(OneToManyMap<java.lang.String,java.lang.String> map)` |
    | `boolean` | `isEmpty()` |
    | `boolean` | `mapIsNullOrEmpty()` public void initialise(Vector keys, Vector values) { this.setMap(); this.generateMap(keys, values); } |
    | `OneToManyMap<T,U>` | `removeEmptyStrings()` |
    | `boolean` | `removeKey(T key)` |
    | `void` | `removeValue(T key, U value)` Remove a value from the key's list of values |
    | `void` | `removeValue(U value)` Remove a value from \*all\* the key's list of values |
    | `void` | `removeValues(java.util.Collection<U> values)` |
    | `java.lang.String` | `toCSV()` Get a list of comma separated [key, value] couples. |
    | `java.lang.String` | `toString()` |

    - ### Methods inherited from class java.util.TreeMap

      `ceilingEntry, ceilingKey, clone, comparator, descendingKeySet, descendingMap, entrySet, firstEntry, firstKey, floorEntry, floorKey, get, headMap, headMap, higherEntry, higherKey, keySet, lastEntry, lastKey, lowerEntry, lowerKey, navigableKeySet, pollFirstEntry, pollLastEntry, put, putAll, remove, size, subMap, subMap, tailMap, tailMap, values`
    - ### Methods inherited from class java.util.AbstractMap

      `equals, hashCode`
    - ### Methods inherited from class java.lang.Object

      `getClass, notify, notifyAll, wait, wait, wait`
    - ### Methods inherited from interface java.util.Map

      `equals, hashCode`

- - ### Constructor Detail


    - #### OneToManyMap

      ```
      public OneToManyMap()
      ```

      Initialise an empty map


    - #### OneToManyMap

      ```
      public OneToManyMap(java.util.Vector<T> keys,
                  java.util.Vector<U> values)
      ```

      Initialise with a list of key-value pairs

      Parameters:
      :   `keys` - the list of keys
      :   `values` - the list of values (the value for each key in the keys list)
  - ### Method Detail


    - #### invertKeysAndValues

      ```
      public static OneToManyMap<java.lang.String,java.lang.String> invertKeysAndValues(OneToManyMap<java.lang.String,java.lang.String> map)
      ```


    - #### removeEmptyStrings

      ```
      public OneToManyMap<T,U> removeEmptyStrings()
      ```


    - #### addAll

      ```
      public void addAll(java.util.Map<T,U> map)
      ```

      Parameters:
      :   `instanceNamesToAttributesStringMap` -


    - #### addAll

      ```
      public void addAll(OneToManyMap<T,U> mapToAdd)
      ```

      Add all elements of a one2many map to another map

      Parameters:
      :   `map` - the map whose elements have to be added


    - #### addKeyAndValue

      ```
      public boolean addKeyAndValue(T key,
                           U value)
      ```

      Add a key-value pair to the map

      Parameters:
      :   `key` - a key
      :   `value` - a value

      Returns:
      :   true if the value was added, false if the key-value pair already
          existed


    - #### clear

      ```
      public void clear()
      ```

      **Specified by:**
      :   `clear` in interface `java.util.Map<T,java.util.Set<U>>`

      **Overrides:**
      :   `clear` in class `java.util.TreeMap<T,java.util.Set<U>>`


    - #### containsKey

      ```
      public boolean containsKey(java.lang.Object key)
      ```

      Checks if the map contains a key

      **Specified by:**
      :   `containsKey` in interface `java.util.Map<T,java.util.Set<U>>`

      **Overrides:**
      :   `containsKey` in class `java.util.TreeMap<T,java.util.Set<U>>`

      Parameters:
      :   `key` -

      Returns:
      :   true if the key exists in the map, false otherwise


    - #### containsKeyValue

      ```
      public boolean containsKeyValue(T key,
                             U value)
      ```

      Checks if the map contains a specific key-value pair

      Parameters:
      :   `key` -
      :   `value` -

      Returns:
      :   true if the key-value exists, false otherwise (false if the key
          or value are null)


    - #### containsValue

      ```
      public boolean containsValue(java.lang.Object value)
      ```

      Checks if the map contains a value

      **Specified by:**
      :   `containsValue` in interface `java.util.Map<T,java.util.Set<U>>`

      **Overrides:**
      :   `containsValue` in class `java.util.TreeMap<T,java.util.Set<U>>`

      Parameters:
      :   `value` - a value

      Returns:
      :   true if the value exists in the map, false otherwise


    - #### deleteKeyValue

      ```
      public boolean deleteKeyValue(T key,
                           U value)
      ```


    - #### generateAKeyValues

      ```
      public java.util.Set<U> generateAKeyValues(T key)
      ```


    - #### getAKeyValues

      ```
      public java.util.Set<U> getAKeyValues(T key)
      ```

      Get the set of unique values for this key. No values can be null. At most
      one of the values can be an empty string.

      Returns:
      :   the set of unique values for this key


    - #### getKeysVectorValuesVector

      ```
      public java.util.Vector<java.util.Vector> getKeysVectorValuesVector()
      ```

      Get 1. a first vector containing each key as many times as values exist
      for the key. 2. a second vector containing one corresponding value for
      each key entry in the first vector. The two vectors together create a 2
      vectors representation of the map (key in vector 1, value in vector 2).
      Example, for a map: [1:{a, b}, 2:{b, c}] the keys vector will contain:
      [1, 1, 2, 2]. While the values vector will contain: [a, b, b, c]. Read
      together, element by element, they give the map couples.

      Returns:
      :   a vector of vectors, with the first vector being the keys vector
          and the second being the values vector.


    - #### getKeysWithValue

      ```
      public java.util.TreeSet<T> getKeysWithValue(U value)
      ```

      Returns all the keys having a certain value

      Parameters:
      :   `value` - a value

      Returns:
      :   the list of keys having that value or null if no keys with that
          value were found


    - #### getKeyValuesPairsSize

      ```
      public int getKeyValuesPairsSize()
      ```

      Get the number of unique key-value pairs in the map. No key can be null.
      At most one of the keys can be an empty string. The values can be empty
      strings.

      Returns:
      :   the number of unique key-value pairs in the map


    - #### getNonEmptyKeys

      ```
      public java.util.Set<T> getNonEmptyKeys()
      ```

      Get the set of unique, non-empty-string keys for this map.

      Returns:
      :   the set of unique, non-empty-string keys for this map


    - #### getNonEmptyKeyValuesPairsSize

      ```
      public int getNonEmptyKeyValuesPairsSize()
      ```

      Get the number of unique, non-empty key-value pairs in the map. No key
      can be null. Empty string keys will not be included, nor will empty
      string values.

      Returns:
      :   the number of unique key-value pairs in the map


    - #### getNonEmptyValues

      ```
      public java.util.Set<U> getNonEmptyValues()
      ```

      Get the set of unique, non-empty-string values for this map.

      Returns:
      :   the set of unique, non-empty-string values for this map


    - #### getRandomValue

      ```
      public U getRandomValue(T key)
      ```

      Get randomly one of the values of this key

      Parameters:
      :   `key` -


    - #### getValues

      ```
      public java.util.Set<U> getValues()
      ```

      Get the set of unique values for this map. (No value can be null if type
      is String, or other class whose comparator does not allow null!). At most
      one value can be an empty string.

      Returns:
      :   the set of unique values for this map


    - #### isEmpty

      ```
      public boolean isEmpty()
      ```

      **Specified by:**
      :   `isEmpty` in interface `java.util.Map<T,java.util.Set<U>>`

      **Overrides:**
      :   `isEmpty` in class `java.util.AbstractMap<T,java.util.Set<U>>`


    - #### mapIsNullOrEmpty

      ```
      public boolean mapIsNullOrEmpty()
      ```

      public void initialise(Vector keys, Vector values) { this.setMap();
      this.generateMap(keys, values);
      }


    - #### removeKey

      ```
      public boolean removeKey(T key)
      ```


    - #### removeValue

      ```
      public void removeValue(T key,
                     U value)
      ```

      Remove a value from the key's list of values

      Parameters:
      :   `key` -
      :   `value` -


    - #### removeValue

      ```
      public void removeValue(U value)
      ```

      Remove a value from \*all\* the key's list of values

      Parameters:
      :   `key` -
      :   `value` -


    - #### removeValues

      ```
      public void removeValues(java.util.Collection<U> values)
      ```

      Parameters:
      :   `queries` -


    - #### toCSV

      ```
      public java.lang.String toCSV()
      ```

      Get a list of comma separated [key, value] couples.

      Returns:
      :   a list of comma separated [key, value] couples.


    - #### toString

      ```
      public java.lang.String toString()
      ```

      **Overrides:**
      :   `toString` in class `java.util.AbstractMap<T,java.util.Set<U>>`


- Overview
- Package
- Class
- Use
- Tree
- Deprecated
- Index
- Help

- Prev Class
- Next Class

- Frames
- No Frames

- All Classes

- Summary:
- Nested |
- Field |
- Constr |
- Method

- Detail:
- Field |
- Constr |
- Method
